# Error estimation on extracorporeal trajectory determination from body scans

**DOI:** 10.1007/s00414-021-02676-y

**Published:** 2021-08-23

**Authors:** F. Riva, U. Buck, K. Buße, R. Hermsen, E. J. A. T. Mattijssen, W. Kerkhoff

**Affiliations:** 1 Centre Universitaire Romand de Medecine Legale Lausanne-Geneva, University Hospital of Lausanne, Lausanne, Switzerland; 2 Ecole Des Sciences Criminelles, Université de Lausanne, Batochime, CH 1015 Lausanne-Dorigny, Switzerland; 3 Institute of Forensic Medicine, University of Bern, Bern, Switzerland; 4 Technical Accident Service, Canton Police Bern, Bern, Switzerland; 5 Netherlands Forensic Institute, The Hague, The Netherlands

**Keywords:** Forensic, Shooting incident reconstruction, Bullet trajectory, Computer Tomography, 3D trajectory reconstruction, Error estimation

## Abstract

This study explores the magnitude of two sources of error that are introduced when extracorporeal bullet trajectories are based on post-mortem computed tomography (PMCT) and/or surface scanning of a body. The first source of error is caused by an altered gravitational pull on soft tissue, which is introduced when a body is scanned in another position than it had when hit. The second source of error is introduced when scanned images are translated into a virtual representation of the victim’s body. To study the combined magnitude of these errors, virtual shooting trajectories with known vertical angles through five “victims” (live test persons) were simulated. The positions of the simulated wounds on the bodies were marked, with the victims in upright positions. Next, the victims were scanned in supine position, using 3D surface scanning, similar to a body’s position when scanned during a PMCT. Seven experts, used to working with 3D data, were asked to determine the bullet trajectories based on the virtual representations of the bodies. The errors between the known and determined trajectories were analysed and discussed. The results of this study give a feel for the magnitude of the introduced errors and can be used to reconstruct actual shooting incidents using PMCT data.

## Introduction

### General introduction

An increasing number of forensic institutes perform post-mortem computed tomography (PMCT) and/or surface scanning prior to autopsy. This allows the acquisition of complete 3D data of the victim’s body. In cases involving bullet trauma, PMCT can be used to document both injuries and foreign materials in the context of firearm injuries. The interpretation as to entrance, exit and trajectory may still pose questions and require autopsy and histology for best approximation. However, integrating these data in a three-dimensional context for the purpose of shooting incident reconstructions is complex and needs further study.

A complete shooting incident reconstruction is a complex task involving several domains. The assemblage of all forensic data and their graphical representation may be time consuming and not always remain free of apparent contradictions. Photographical documentation, supplemented by three-dimensional techniques like photogrammetry and laser scanning [1–9], can be used for this task. These latter techniques allow to reconstruct and represent a crime scene in a virtual 3D space. On basis of these crime scene and integrated medico-legal findings, shooting trajectories may be approximated [10, 11]. The wound channel information extrapolated from PMCT and/or surface scanning coupled to (and corroborated with) the autopsy findings are often integrated in such a three-dimensional crime scene in the form of so-called 3D mannequins, virtual dummies or Bipeds [12–15] as will be described in more detail below. For consistency, we will use the term Bipeds in the current paper. The body proportions and dimensions of a Biped correspond to those of the victim [13, 16–18]. A Biped can be used in 3D animations by moving its position and posture, while ensuring the anatomical relationship between the different articulations and body parts [17]. The danger of introducing bias into a legal procedure with this type of representation is a discussion in itself [19] but is left out of scope in this study. Another aspect that is left out of scope is the incertitude of bullet deflection. The trajectory of a bullet that perforated a victim can sometimes be reconstructed from defects in fixed objects towards the estimated location of the exit wound. Several studies in the literature [20–23] can be used for this purpose, including the incertitude involved. If the trajectory is to be reconstructed further, from the victim towards the estimated location of a shooter, more incertitude is introduced by possible bullet deflection in the victim’s body. When the trajectory between the shooter and the entry wound is established by connecting the entry to the exit wound [12], the possible deviation of the projectile in the intermediate tissues has to be taken into account [24–28]. When no data about this deviation is available, ballistic tests will have to be performed [15]. These possible sources of error are also left out of scope in the current study. Finally, the current study can only be used to assess the trajectories of perforating bullets that produce both an entry and exit wound. Bullets that penetrate without exiting are also left out of scope.

### The scope of the current study

The current study was designed to assess two sources of error that are introduced when extracorporeal bullet trajectories from perforating bullets are assessed from PMCT data, coupled or not with a surface scan of the body. The first source of error is introduced when a body is scanned in another position than it had when hit. Victims of shooting incidents will often have an upright, vertical position when hit, but will be scanned lying down when deceased. This alters the direction of gravitational pull on the body. The effect of this altered pull, in terms of tissue displacement, will be aggravated when muscle tension ends after death. The second source of error is introduced when PMCT images are translated into a Biped, which is a simplified, virtual representation of a body and not a full anatomically correct copy.

In order to investigate the abovementioned error sources in a simplified and controlled environment, the study protocol included the following: (1) three simplified mock crime scenes, each consisting of a single virtual bullet trajectory; (2) a victim (living test person) positioned and scanned in this trajectory in an upright position; and (3) a scanning procedure of the same victim in supine position, producing a partial substitute of actual PMCT data. This procedure is based on the following assumptions: (a) a straight bullet trajectory into the body has been considered, which in any real case may not be a given [25–27, 29]; (b) the victims assumed a motionless, standing position; (c) only the aspect of metric body tissues shift between standing and supine position and the effects on the vertical angle estimation of a bullet trajectory are examined.

## Materials and methods

### Crime scene

**Fig. 1:**
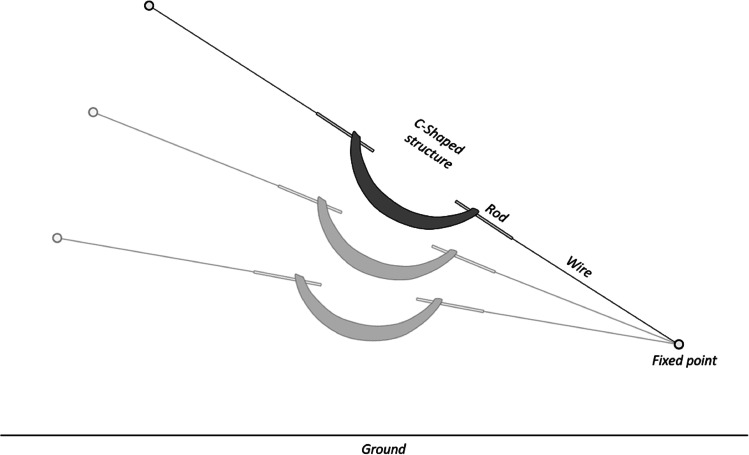
Scheme representing the concept of mock crime scene with a simulated straight bullet trajectory. No dimension proportionality is respected in this example

The simplified “crime scene” consisted of a single mock bullet trajectory, based on a wire strung tightly between two fixed points in a small room. The line held a C-shaped interruption in the middle, with two hinged metal rods on each end. The line represents a simulated straight bullet trajectory through the body of a victim, “frozen in time”. By modifying the fixed points in the room, it is possible to change the elevation angle of the simulated bullet trajectory. Three scenes with different shooting angles, named A, B and C, were prepared (see Fig. 1).

### Crime scene scanning and ground truth determination

**Fig. 2:**
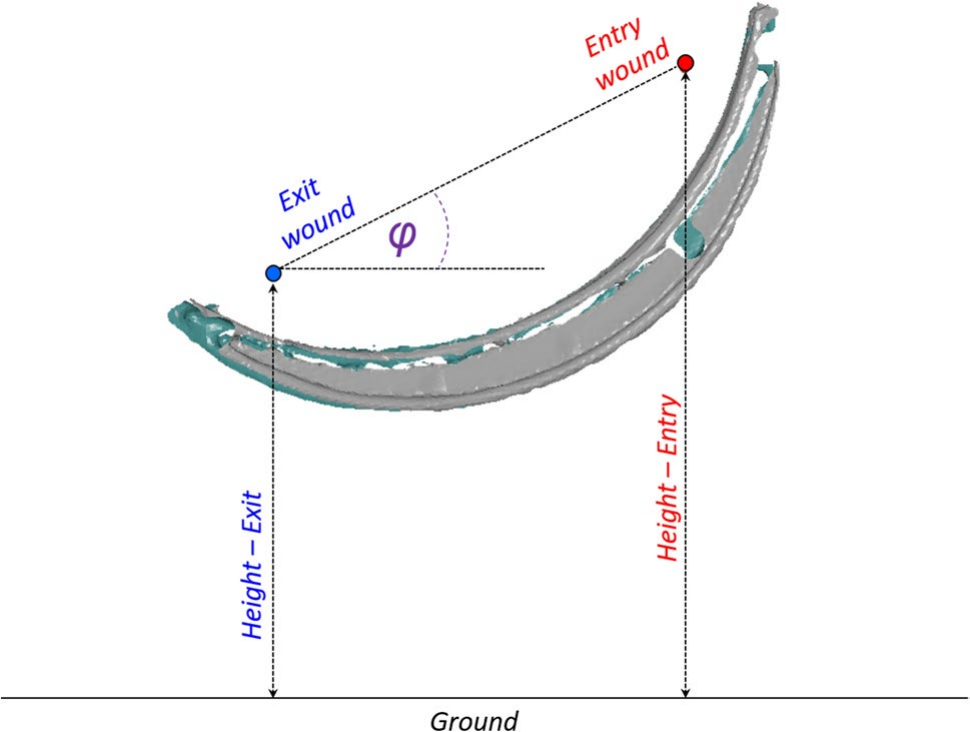
Example of shooting angle measurement based on the scanned scene. The red dot (right) and the blue dot (left) represent respectively the entry and the exit wounds. Their height and vertical trajectory angle φ relative to the ground’s surface can be measured in the GOM Inspect software

The trajectory (ground truth) was determined by measuring the vertical shooting angle φ (elevation) and the position of the tips of the rods (entry and exit wounds in Fig. 2) with respect to the coordinate system of the scene (ground). All measurements have been performed using the 3D software GOM Inspect software (GOM, Braunschweig, Germany).

The measurements were performed by scanning the scene with a Creaform Go!SCAN 50 hand-held scanner [29]. This scanner is based on the emission of a structured white light pattern. Two cameras observe the distortion of the pattern on the scanned object. A third camera records the colour information of the points. The scanner works with a rate of 550,000 measurements per second and a scanning area of 380 × 380 mm with a resolution of up to 0.5 mm and a point accuracy of up to 0.1 mm. The surface was captured while moving the hand-held scanner over the object [29]. Black and white stickers, called positioning targets, were put on various parts of the crime scene surface, including the tips of the metal rods. These positioning targets are easily recognised by the scanner software contributing to the precision of the scan. The accuracy between two positioning targets separated by a distance of 5 m from each other is ± 0.43 mm [29]. The resulting scan is then exported in.STL format and imported in GOM Inspect software to be able to measure the relevant dimensions. Only one operator was responsible for the set-up and the measurements of the three scenes, avoiding possible between-operator differences. The shooting angle is represented only by its elevation component, omitting the azimuth component. For the assessment of elevation, the assumption of a level surface area, orthogonal to gravity, suffices. Azimuth assessment would require some other form of reference for the body in 3D space, which is much harder to provide in an un-ambiguous way. Consequently, the most relevant information for each crime scene are the shooting angle, the entry wound’s height and the exit wound’s height; these values are listed in Table 1.

**Table 1:** Relevant information related to each simulated crime scene

Scene	Entry wound height	Exit wound height	Shooting angle
	[cm]	[cm]	[°]
A	107.4	95.9	-26.1
B	125.5	115.4	-22.6
C	86.4	80.4	-13.3

### Victim scanning

Five persons were used to simulate the victims. The set-up of scene A was used three times, with victims 1, 2 and 3. For each victim a separate scan was performed. Victim 4 was scanned once in scene B and victim 5 were scanned into scene C. The size, gender and crime scene position of each victim are summarised in Table 2.

**Table 2:** Victim (test persons) information

Scene	Victim #	Length	Gender	Body mass index (BMI)	Position
		[cm]			
A	1	182.0	M	28.7	Standing, straight
A	2	163.0	F	19.6	Standing, straight
A	3	179.0	M	24.3	Standing, knees bent slightly
B	4	179.5	M	20.8	Standing
C	5	170.0	M	18.7	Sitting on a chair

**Fig. 3:**
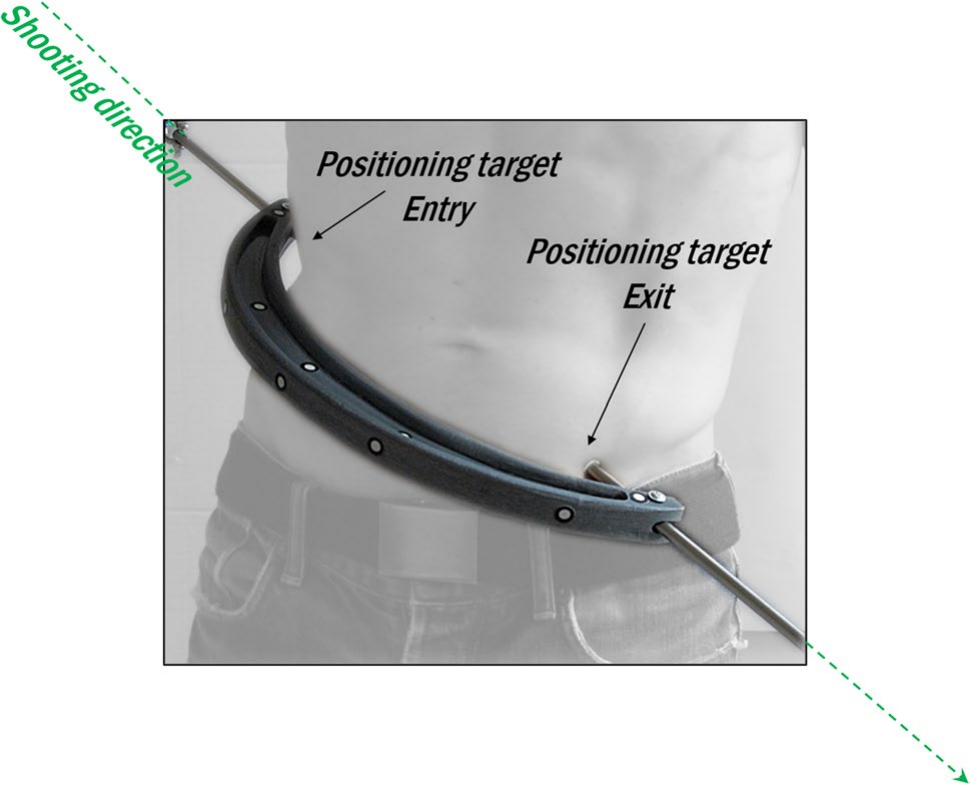
Image illustrating the position of a “victim” in the C-shaped structure. The tips of the two rods, touching the victims skin, mark the entry and exit wound of the virtual bullet trajectory

Each potential victim was asked to take off shoes and t-shirt, assume a specific position (see Table 2) and fit his or her body in the C-shaped interruption in such a way that the skin was in light contact with both metal rods’ tips (see Fig. 3). Shoes were not considered in the test in order to avoid the introduction of additional parameters which could make the result interpretation harder and deviate from the main aim of the research. This information was available to the experts (see “Biped creation and trajectory assessment” section).

Two positioning targets were attached to the victim’s skin on the points of contact with the metal rods (Fig. 3). The highest and lowest contact points represent the entry and the exit wounds respectively, resulting in a downward trajectory with a negative angle for all scenes. As can be seen in Table 2, the victim in crime scene C was sitting. This victim was asked to sit with an upright torso when the entry and exit wounds were marked. The dimensions of the chair were also scanned, with a person sitting to compensate for the deformation of the chair by the body weight.

After marking the simulated entry and exit wounds with positioning targets on the skin, the victims were scanned in supine position (lying face upwards, simulating PMCT). It is undesirable to use computed tomography (CT) on living persons without medical necessity. The best found alternative was an outer body scan with the Go!SCAN 50 hand-held scanner. One drawback of this method (see “Skeleton data” section) is the fact that an outer body scan does not provide the skeleton data that are available with a PMCT scan. A rigid stretcher was used to accommodate the victims wearing only pants or underwear in a supine position while allowing the maximum amount of body surface area when scanned. Contrarily to best practice in casework, only the supine position has been considered because it was not feasible to scan the entry and exit wounds simultaneously in supine and prone position.

The scanner recognises the entry and the exit wounds on the victim’s body by means of the positioning targets on the skin. The result is a surface scan of the victim’s body with the wounds’ coordinates (x,y,z), which can be exported in a.STL and.TXT file.

To have a better overview on the different sources of error, the Euclidean distance between the entry wound and the exit wound on the crime scene (ground truth), namely the distance between the blue dot and the red dot in Fig. 2, is compared to the Euclidean distance between the entry wound and the exit wound on the scan performed on the victim in supine position. The difference between these two values represents the combined influence of the changes caused by the gravitational pull on soft tissue (when a body is scanned in another position than it had when hit) and the difference in body posture (straight or slumped torso).

### Biped creation and trajectory assessment

**Fig. 4:**
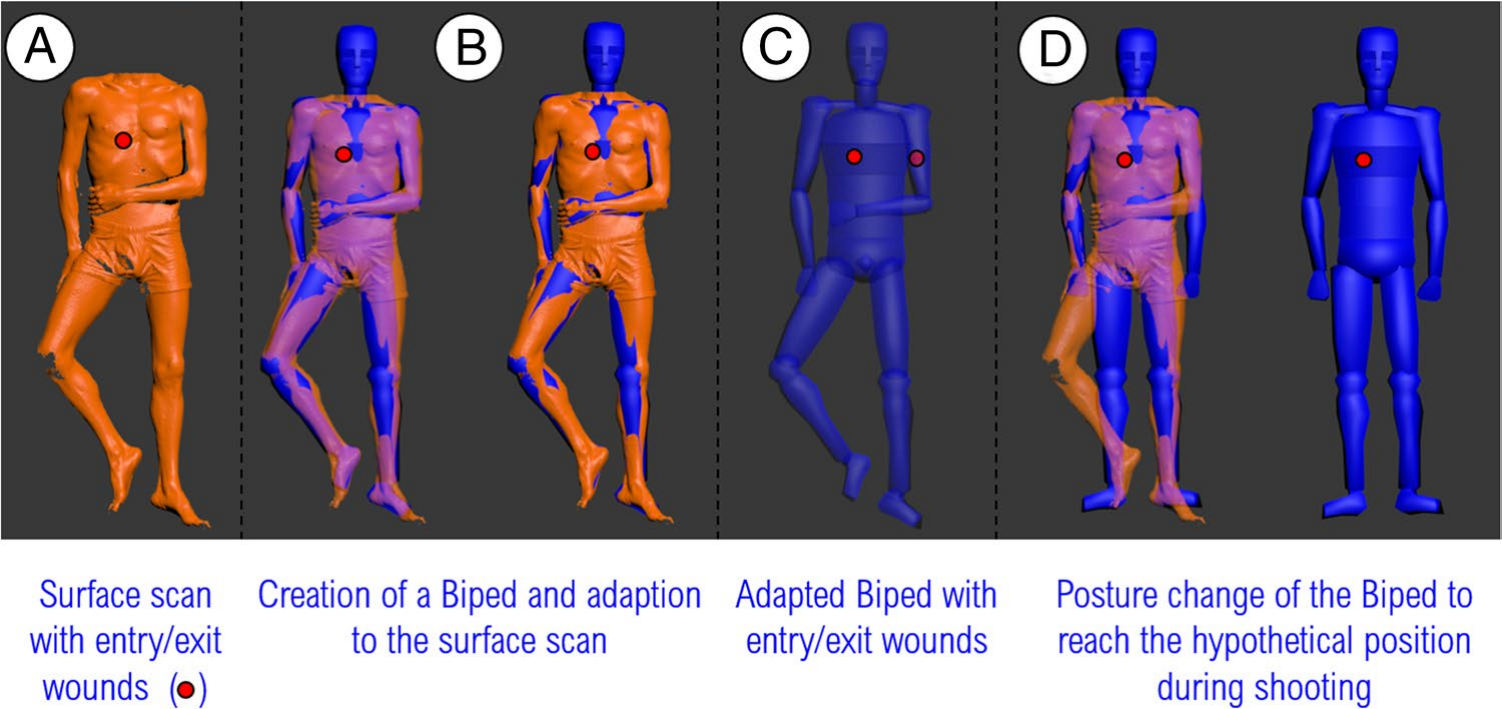
Images illustrating the Biped adaption to the victim’s body size. The red dots represent the entry and the exit wounds. But the entry wound is only visible in illustration C where the Biped is displayed with the opacity of 50%

The scans of the five victims in the form of.STL files, the coordinates of the entry and exit wounds on the skin of the victims (.TXT file), as well as the length of the victims were given to seven 3D forensic experts from two different countries, working in five different laboratories. All the experts worked on the same data, with the same information and using the same software, namely 3ds Max (© 2021 Autodesk Inc.). The experts were asked:

- To model the victim (Fig. 4A) by creating a Biped from the body scan (Fig. 4B) according to the procedure described by Buck et al., 2013. The procedure includes the adaption of the body’s size and proportions and incorporation of the entry and exit wounds to the Biped (Fig. 4C).
- To position the Biped into the scene. The experts were provided with limited information to enable them to choose a hypothetical position (Fig. 4D) of the victim. This information was that victims 1 to 4 (scenes A and B) were probably standing and that victim 5 (scene C) was sitting on a chair. The scan of the chair (see “Victim scanning” section) from crime scene C was provided.
- To determine the trajectory by drawing a straight line through the entry and the exit wounds. The results were reported by the experts in the form of a vertical trajectory angle (elevation angle).

The incorporation of the entry and exit wounds from the scan to the Biped (Fig. 4B, C), followed by the change in Biped’s position (Fig. 4D), can introduce an additional error. To track this error, the Euclidean distance between the entry and exit wounds after the scan in supine position is compared to the same distance after the Biped assumed the final position.

### Skeleton data

Adapting a Biped’s dimensions to those of a victim’s body is normally facilitated by the presence of the skeleton structure. Skeleton data is available with a CT scan but not with the surface scans used in this study. In order to explore the potential influence of this factor, an additional dataset was provided to the experts. This dataset was taken from an actual case involving a deceased victim. This victim was shot once through the upper part of the left leg. The projectile perforated the soft tissues without hitting bone. For this dataset, the experts had to provide the shooting angle estimation by adapting the Biped without the skeleton data (only surface scan) and with skeleton data (PMCT with outer scan). For both angle estimations, the positions of the entry and exit wounds were provided in relationship with the surface scan.

## Results

This section is structured sequentially in order to provide a better overview on which kind of error takes place during the abovementioned procedure. The whole procedure is analysed according to the following steps: (a) differences between scene and scan in a supine position (see “From scene to scan in supine position” section); (b) error during the adaption of the Biped to the scan and change in Biped’s position (see “Error during Biped adaption” section); (c) error in shooting angle estimation starting from supine scans (see “Shooting angle estimation” section); (d) influence of the skeleton (see “Influence of the skeleton” section).

### From scene to scan in supine position

The difference in Euclidean distance does not provide accurate information regarding the direction of tissues/skin displacement between the position on the scene and the supine position on the stretcher. However, the results demonstrate that a change in position takes place. Further analysis should be performed to investigate the source of such displacement, namely the gravitational pull on soft tissue, that is introduced when a body is scanned in another position than it had when hit, and/or the difference in body posture.

### Error during Biped adaption

The results show that this step in the procedure also introduces a small error, which contributes to the final error related to the shooting angle estimation (see “Shooting angle estimation” section). In general, the error introduced in this phase is smaller than that in the previous phase (see Tables 3 and 4).

**Table 3:** Entry-exit wounds Euclidean distance comparison between data recorded on the scene (ground truth) and the data from the scan in supine position

Ground truth on the scene			From scan in supine position				
Scene	Position in the scene	Euclidean distance Entry-Exit	Person #	Position during scan	Euclidean distance Entry-Exit	Difference	BMI
A	Standing	261.2 mm	1	Supine	264.0 mm	− 2.7 mm	28.7
A	Standing	261.2 mm	2	Supine	247.8 mm	13.4 mm	19.6
A	Standing bent knees	261.2 mm	3	Supine	286.3 mm	− 25.1 mm	24.3
B	Standing	261.8 mm	4	Supine	279.9 mm	− 18.1 mm	20.8
C	Sitting straight	260.2 mm	5	Supine	295.3 mm	− 35.1 mm	18.7

**Table 4:** Entry-exit wounds Euclidean distance comparison between data from scan in supine position and after Biped adaption and posture modification (mean and SD seven experts)

From scan in supine position			After Biped adaption		
Person #	Position during scan	Euclidean distance entry-exit [mm]	Expert’s hypothetical position (Biped position)	Euclidean distance entry-exit [mm]	Difference [mm]
				Mean ± SD	Mean ± SD
1	Supine	264	Standing	264.2 ± 0.2	− 0.2 ± 0.2
2	Supine	247.8	Standing	247.0 ± 2.1	0.8 ± 2.1
3	Supine	286.3	Standing	282.0 ± 10.0	4.3 ± 10.0
4	Supine	279.9	Standing	273.0 ± 5.5	6.9 ± 5.5
5	Supine	295.3	Sitting and leaning against backrest	290.3 ± 0.2	5.3 ± 0.2

### Shooting angle estimation

The means and standard deviations (SD) of the results provided by the seven experts for the five simulations are summarised in Table 5.

**Table 5:** Results obtained by the seven experts during the shooting angle estimation for the five “victims”

Scene	Person #	Gender	Actual position	Expert’s hypothetical position	Actual shooting angle [°]	Estimated angle [°]	△Angle [°]
						Mean ± SD	Mean ± SD
A	1	M	Standing	Standing	− 26.1	− 27.5 ± 2.1	− 1.4 ± 2.4
A	2	F	Standing	Standing		− 28.7 ± 0.8	− 3.6 ± 3.6
A	3	M	Standing bent knees	Standing		− 23.0 ± 3.2	3.1 ± 4.0
B	4	M	Standing	Standing	− 22.6	− 22.1 ± 1.8	0.5 ± 1.7
C	5	M	Sitting straight	Sitting and leaning against backrest	− 13.3	2.5 ± 4.4	15.8 ± 16.3

### Influence of the skeleton

The mean and standard deviation (SD) of the results provided by the experts with and without skeleton data are summarised in Table 6.

**Table 6:** Results obtained by the experts during the evaluation of the influence of the skeleton for the shooting angle estimation by the mean of a Biped adaption

Biped adaption	Gender	Actual position	Estimated angle [°]
			Mean ± SD
Without skeleton	M	Unknown	− 26.9 ± 1.5
With skeleton			− 25.3 ± 0.6

A two-sample statistical *t*-test was performed on the two distributions of estimated angles with a significant level of 0.05. The results show that, although the difference is small, the means of the two data sets are significantly different (*p* = 0.019).

## Discussion

The results of this exploratory study show that the estimated angles (see “From scene to scan in supine position” section) were reasonably consistent between the experts, with a standard deviation ranging from 0.8° to 4.4°, depending on the scenario. The mean differences between the estimated and actual angles ranged from − 3.6° to + 3.1° for the standing victims. Considering the differences in the Euclidean distances, the main contributing factor is the altered gravitational pull and/or change of body position between the moment of the “shot” and the moment of scanning. The contribution of the Bided adaption was smaller. The definition “standing” leaves a range of minimal posture differences open (e.g., spine, shoulders and knees straight or relaxed). This uncertainty can affect the accuracy of the estimations. As an example, the standard deviation of the seven estimated angles for victim 2 was fairly small (0.8°), but the difference between the estimated and actual angle was fairly large (3.6°). For this victim, the experts seem to have consistently placed the Biped in a more forced, upright position than the actual relaxed position she had while being scanned. For victim 5, who was scanned in a sitting position, the observed mean difference between estimated and actual angle was + 15.8°. The main reason for this greater difference was the erroneous assumption by the experts that victim 5 was leaning against the backrest of the chair, while in fact he was sitting upright when scanned. This scenario was included solely to illustrate the fact that erroneous body posture estimations will greatly affect the accuracy of estimated trajectory angles.

### Limitations in generalising these results

By modelling the extracorporeal trajectory as a straight line between the (mock) entry and exit wounds in this study, any intracorporeal bullet deviation was not considered. Assuming a bullet to follow a straight intracorporeal trajectory is often not realistic, because many calibres and/or projectiles are known to deviate from their original path in soft tissues [24]. Contact with bone might affect the path of a bullet even more. Depending on the type of projectile and length of a wound channel [15, 25–28], an additional error must be considered when trajectory reconstructions are performed.

Another aspect that must be taken into account might be the body mass index (BMI) of the victim. The body size (corpulence) differed among the victims 1–5 (see Tables 2 and 3), but none of them was morbidly obese (BMI greater than 40). Different amounts of subcutaneous fat might result in differences in soft tissue displacement by gravity, and therefore the location of entry and exit wounds, when the position of a body is altered from upright to supine. The results of the current study might not be applicable to victims with a high BMI.

Another limitation of this study is the fact that the victims were motionless (standing or siting still) when scanned at the crime scenes. In actual shooting incidents, victims might move rapidly as they attempt to fight, flee or take cover in the face of an imminent threat. This motion might lead to distorted body postures and/or momentarily shifts of skin and soft tissue. However, movement does not always apply. Victims can be hit by surprise or being executed while motionless. Whether motion can or cannot be assumed must be considered from case to case.

Finally, the results of this study might have been influenced by the fact that the scans lack the skeleton data that are normally available with PMCT data. This might have negatively affected the accuracy of the Biped. That availability of skeleton data can have an effect was illustrated by the test results shown in “Influence of the skeleton” section. Since the data for this test were taken from a shooting incident, the actual shooting angle (ground truth) is not known and can therefore not be compared to the estimated angles. However, the results do show a small, but significant difference between the mean estimated angles, made without and with skeleton data by the seven experts.

## Conclusions

Extracorporeal trajectory reconstructions, based on intracorporeal findings from PMCT and/or surface scanning, can be a useful tool in crime scene analysis. In the current study, 3D trajectory reconstructions using Bipeds lead to fairly consistent results between experts. The estimated trajectories were also close to the actual known trajectories, when the experts made correct assumptions on the victim’s body postures. As was expected, erroneous assumptions regarding body postures lead to differences between the estimated and actual trajectory angles.

The presented results, while acquired in a concise study, give a feel for the magnitude of the errors introduced by soft tissue displacement and Biped creation from scan data. The results from the current study can be used in reconstructing actual shooting incidents, when the scope and limitations of the study are taken into account.

## Data Availability

Under request all the data can be asked to the corresponding author.
